# Benzophenone Derivatives from an Algal-Endophytic Isolate of *Penicillium chrysogenum* and Their Cytotoxicity

**DOI:** 10.3390/molecules23123378

**Published:** 2018-12-19

**Authors:** Dong-Lin Zhao, Xiao-Long Yuan, Yong-Mei Du, Zhong-Feng Zhang, Peng Zhang

**Affiliations:** Tobacco Research Institute, Chinese Academy of Agricultural Sciences, Qingdao, Shandong 266101, China; zhaodonglin@caas.cn (D.-L.Z.); rayrock@126.com (X.-L.Y.); duyongmei@caas.cn (Y.-M.D.); zhangzhongfeng@caas.cn (Z.-F.Z.)

**Keywords:** marine algal-derived fungi, *Penicillium chrysogenum*, secondary metabolites, polyketides, benzophenone derivatives, cytotoxicity

## Abstract

Chromatographic separation of a marine algal-derived endophytic fungus *Penicillium chrysogenum* AD-1540, which was isolated from the inner tissue of the marine red alga *Grateloupia turuturu*, yielded two new benzophenone derivatives, chryxanthones A and B (compounds **1** and **2**, respectively). Their structures were undoubtedly determined by comprehensive analysis of spectroscopic data (1D/2D NMR and HRESIMS). The relative and absolute configurations were assigned by analysis of the coupling constants and time-dependent density functional theory (TDDFT) calculations of their electronic circular dichroism (ECD) spectra, respectively. Both compounds possessed an unusual dihydropyran ring (ring D) fused to an aromatic ring, rather than the commonly occurring prenyl moiety, and a plausible biosynthetic pathway was postulated. The cytotoxicities of compounds **1** and **2** were evaluated against six human cell lines, and both of the compounds demonstrated weak to moderate cytotoxicities with IC_50_ values ranging from 20.4 to 46.4 μM. These new compounds further demonstrate the potential of marine-derived fungi as an untapped source of pharmaceutical components with unique properties that could be developed as drug candidates.

## 1. Introduction

Marine-derived fungi, which are recognized as frontier resources for drug candidate discovery, are prolific sources of bioactive secondary metabolites with unique structures [1]. The unique features of the marine environment (e.g., hypersaline, high-pressure, anoxic, and disphotic conditions) result in distinct structures of the secondary metabolites secreted by marine-derived fungi from those produced by terrestrial microorganisms [2]. Chemical investigations of them have resulted in the isolation and identification of numerous polyketides, terpenes, peptides, steroids, and alkaloids, many of which exhibited pharmaceutically relevant bioactivities. Thus, secondary metabolites isolated from marine-derived fungi are considered as an untapped resource in the search for novel lead compounds and/or new drugs [3].

**Figure 1:**
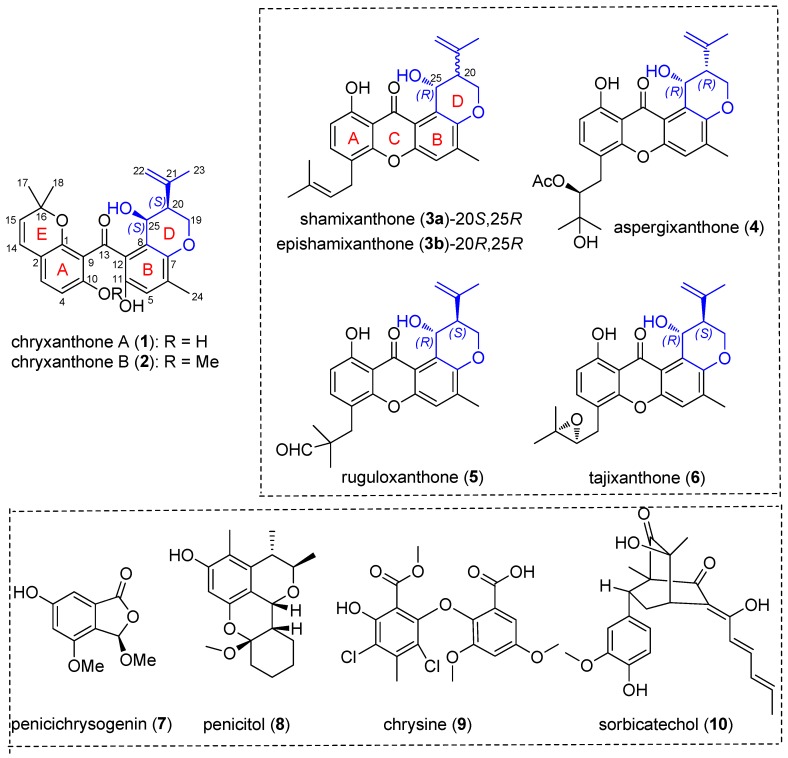
The structures of chryxanthone A (**1**) and B (**2**) and previously reported compounds, shamixanthone (**3a**), epishamixanthone (**3b**), aspergixanthone (**4**), ruguloxanthone (**5**), tajixanthone (**6**), penicichrysogenins (**7**), penicitols (**8**), chrysines (**9**), and sorbicillinoids (**10**).

The fungal strain *Penicillium chrysogenum* is a rich source of several structurally diverse polyketides (anthraquinones, phenols, xanthones, macrolides, etc.), including penicichrysogenins with nitric oxide inhibitory activity [4], penicitols with cytotoxic activity [5], chrysines with α-glucosidase inhibitory activity [6], and sorbicillinoids with antiviral activity [7] (Figure 1). During our unremitting search for novel natural products from marine-derived fungi [8,9], an endophytic strain *P. chrysogenum* isolated from the marine red alga *Grateloupia turuturu* was chemically investigated. Consequently, two new benzophenone derivatives, namely, chryxanthone A (**1**) and B (**2**), were obtained. Both compounds have an unusual dihydropyran ring incorporated in the structure (ring D), which is fused to an aromatic ring and an open ring (ring C) (Figure 1), proving to be unusual members of benzophenone derivatives in structurally related xanthones. To the best of our knowledge, only a few related prenylxanthones, belonging to the families of shamixanthones, tajixanthones, ruguloxanthones, and aspergixanthones, have been previously reported (Figure 1) [10,11,12,13,14]. In this study, we report the details of the isolation, structure elucidation, and cytotoxic activities of these new polyketides, providing a foundation for further exploration of their pharmaceutical properties as novel drug candidates.

## 2. Results and Discussion

### 2.1. Structural Elucidation

**Figure 2:**
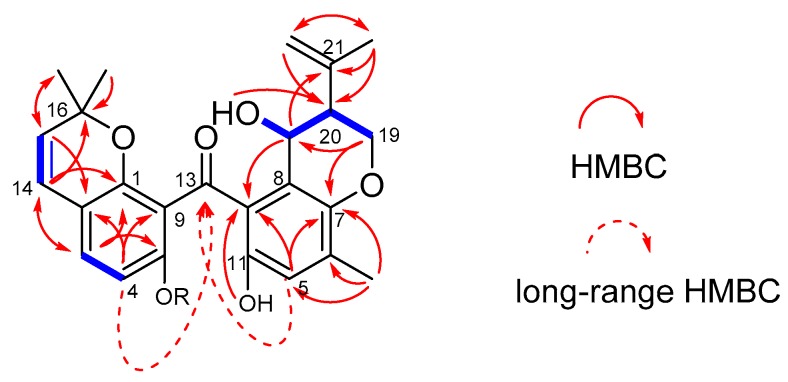
COSY (heavy blue lines) and selected HMBC (red arrows) correlations for **1** and/or **2**.

Compound **1** was obtained as yellowish oil. Its molecular formula, C_25_H_26_O_6_, was determined based on a prominent pseudo-molecular ion peak at *m*/*z* 421.1653 [M − H]^−^ in the high-resolution electrospray ionization mass spectroscopy (HRESIMS) spectrum (Figure S1). The ^1^H-NMR spectrum (Table 1; Figure S2) revealed signals attributable to five aromatic/olefinic protons (δ_H_ 7.12 (d, *J* = 8.4 Hz, H-3), 6.39 (d, *J* = 8.4 Hz, H-4), 6.58 (s, H-5), 6.26 (d, *J* = 9.8 Hz, H-14), and 5.44 (d, *J* = 9.8 Hz, H-15)), two methine protons (δ_H_ 2.34 (H-20) and one oxygenated at δ_H_ 4.53 (H-25)), two methylene protons with one terminal olefinic signal (δ_H_ 4.83 (m, H-22a) and δ_H_ 4.61 (m, H-22b)) and one oxygenated signal (δ_H_ 4.18 (m, H-19a) and δ_H_ 4.13 (m, H-19b)), four singlet methyl groups (δ_H_ 0.98 (s, H-17), 0.92 (s, H-18), 1.70 (s, H-23), and 2.10 (s, H-24)), and two exchangeable protons resonating at δ_H_ 12.20 (s, OH-10) and 8.88 (s, OH-11). In the ^13^C-NMR spectrum (Table 1; Figure S3), 25 resonances were clearly observed, which were classified as one ketone carbonyl (δ_C_ 200.6, C-13), 16 aromatic/olefinic carbons (including two benzene rings, one cis double bond, and one terminal double bond), two sp^3^ methines (one of which was oxygenated), one oxygenated methylene, and four methyls. The ^1^H–^1^H COSY spectrum (Figure S4) showed clear correlations between H-19/H-20, H-20/H-25, and H-25/OH-25, which can be deduced to a −OCH_2_CHCHOH spin system. The dihydropyran ring D was constructed by HMBC correlations (Figure 2) from H-19 to C-7, from H-20 to C-8, and from H-25 to C-7 and C-8. Further HMBCs from H-23 and H-22 to C-20/C-21 indicated an isopropenyl moiety located at C-20. Ring E (2,2-dimethyl-2*H*-chromone moiety) and the benzene ring A were also deduced by some key HMBC correlations, as shown in Figure 2A. The above substructures (rings A and B) must connect via a ketone carbonyl group (C-13), as evidence from the observation of long-range HMBC correlations from H-4 and H-5 to C-13, which can also be detected by Wu et al. [15]. Compound **1** was considered to be a benzophenone derivative, and it was given the trivial name of chryxanthone A.

The relative configuration of compound **1** was assigned by comparison of the pattern of the coupling constants with data from the literature. Previous reports on relative stereochemistry of D ring indicated both syn- and anti-configurations, and they were distinguished based on different forms of coupling constants. When the coupling constant of H-19b and H-20 is small, it is suggested that isopropenyl group is pseudo-axial oriented and the OH-25 group and the isopropenyl group therefore possesses anti stereochemistry. Examples of this kind of situation included shamixanthone (*J*_H19b–H20_ = 2.8 Hz) [10], tajixanthone derivatives (*J*_H19b–H20_ = 2.8 Hz) [12], and aspergixanthones J–K (*J*_H19b–H20_ = 3.0 Hz) [14]. Conversely, if the coupling constant of H-19b and H-20 is a large value, the isopropenyl group on C-20 is oriented equatorial and it means that OH-25 and the isopropenyl group has a *syn* relationship, such as epitajixanthone hydrate (*J*_H19b–H20_ = 11.8 Hz) [12], aspergixanthone I (*J*_H19b–H20_ = 12.0 Hz) [14], and epishamixanthone (*J*_H19b–H20_ = 11.0 Hz) [16]. In this study, the pattern of the coupling constants of compound **1** was in accordance with the relevant characteristics of previously reported related compounds, and the large coupling constant of H-19b and H-20 (*J* = 11.0 Hz) indicated a syn relationship.

**Figure 3:**
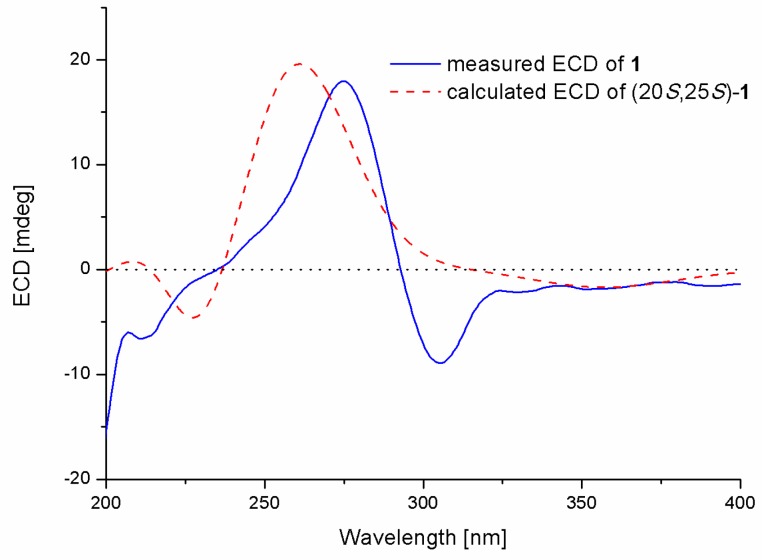
Experimental and calculated (electronic circular dichroism) ECD spectra of compound **1** in MeOH (Blue solid line and red dotted line represent measured and calculated ECD spectra, respectively; X- and Y-axis represent wavelength and ECD value.).

The absolute configuration of compound **1** was determined by comparison of its electronic circular dichroism (ECD spectrum) with that of related compounds. Zhu et al. reported that the Cotton effects (CE) at 250–300 nm and 300–350 nm are diagnostic features of a dihydropyran ring system, and the negative CE at approximately 280 nm and positive CE at 310 nm are responsible for the 25*R* configuration [13,14]. The ECD spectrum of compound **1** showed positive CE at 282 nm and negative CE at 328 nm, which was contrary to that of aspergixanthones [13,14]; thus, the absolute configuration of compound **1** was proposed as 25*S*. The absolute configuration of compound **1** was further determined by the time-dependent density functional theory (TDDFT)-ECD calculation in Gaussian 09 (revision D.01, Gaussian, Inc., Wallingford, CT, USA) [17] at the PBE0/TZVP level to generate the calculated ECD spectrum of compound **1**. The gas-phase B3LYP/6-31G(d) re-optimization of the initial Merck molecular force field (MMFF) conformers of (20*S*,25*S*)-**1** afforded 4 conformers above 2% population (Figure S15). The experimental ECD spectrum of compound **1** showed good agreement with that calculated for (20*S*, 25*S*)-**1** (Figure 3), confirming the absolute structure of compound **1**.

**Figure 4:**
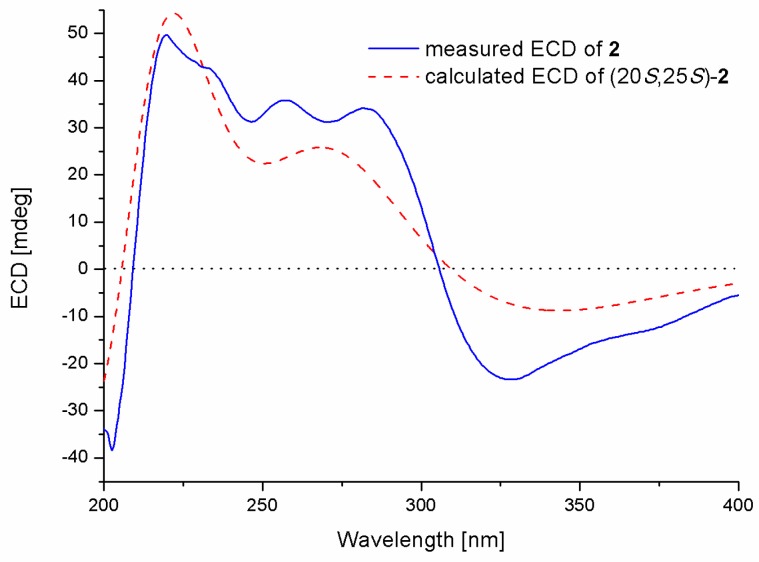
Experimental and calculated ECD spectra of compound **2** in MeOH (Blue solid line and red dotted line represent measured and calculated ECD spectra, respectively; X- and Y-axis represent wavelength and ECD value.).

Compound **2** was also isolated as yellowish syrup, with the molecular formula C_26_H_28_O_6_ determined by the HRESIMS spectrum (*m*/*z* 435.1814 [M − H]^−^, calcd for C_26_H_27_O_6_, 435.1813) (Figure S8). Compound **2** was elucidated as the C-10 *O*-methyl derivative of compound **1**, as evident from the appearance of an additional methoxy group (*δ*_H_/*δ*_C_ 3.69/56.3) in compound **2**, as well as the key HMBC correlation from OCH_3_-10 to C-10 (*δ*_C_ 158.1) (Figure S13). The similarities of the coupling constants and plausible biosynthetic pathways between compounds **1** and **2** suggested the same configurations for the two compounds. Moreover, the experimental ECD spectrum for compound **2** showed good agreement with that calculated for (20*S*, 25*S*)-2 (Figure 4). A trivial name, chryxanthone B, was designated for this compound.

Although naturally occurring benzophenone derivatives are common in nature, these compounds incorporating a dihydropyran ring (ring D) in the structures are particularly unusual. To date, only about 30 related compounds belonging to the families of shamixanthones, tajixanthones, ruguloxanthones, and aspergixanthones have been reported. In this study, two new benzophenone derivatives with an unusual dihydropyran ring were obtained. The new compounds may be biosynthesized from the same pathways through multiple modifications, such as hydroxylation, prenylation, cyclodehydration, cyclization, and methylation (Scheme S1) [18,19,20].

### 2.2. Cytotoxic Activity of the New Compounds

Compounds **1** and **2** isolated in this study were submitted to a Cell Counting Kit-8 (CCK-8) (Dojindo, Kumamoto, Japan) colorimetric assay with six human tumor cell lines (A549, BT-549, HeLa, HepG2, MCF-7, and THP-1) to assess their cytotoxicities. Compound **1** showed moderate cytotoxicity against the BT-549 and HeLa cell lines, with IC_50_ values of 20.4 and 23.5 μM, respectively, while compound **2** displayed a selective growth-inhibitory effect on the A549 cell line with an IC_50_ value of 20.4 μM (Table 2).

## 3. Materials and Methods

### 3.1. General Experimental Procedures

Optical rotations were recorded on a Jasco P-1020 digital polarimeter (Jasco, Tokyo, Japan) at 25 °C. The UV spectra were obtained on a Shimadzu UV-2700 spectrophotometer (Shimadzu, Kyoto, Japan). ECD spectra were obtained with a Jasco J-815-150S circular dichroism spectrometer (Jasco, Tokyo, Japan). The ^1^H, ^13^C, and 2D NMR experiments were performed on an Agilent DD2 spectrometer (500 and 125 MHz, respectively) (Agilent, Santa Clara, CA, USA). The HRESIMS data were obtained on a Thermo Scientific LTQ Orbitrap XL spectrometer (Thermo Scientific, Waltham, MA, USA). Column chromatography (CC) was performed using silica gel (Qingdao Haiyang Chemical Factory, Qingdao, China) and Lobar LiChroprep RP-18 (Merck, Darmstadt, Germany).

### 3.2. Fungal Material and Fermentation

The fungus AD-1540 used in this study was initially isolated from the fresh inner tissue of the red marine alga *Grateloupia turuturu*, collected in August 2016 from Qingdao, China. The fungal strain was isolated and purified according to previously reported methods [21]. It was identified as *Penicillium chrysogenum* (GenBank accession number MH997569) following standard procedures [21].

The fungal strain was statically fermented on a sterilized solid medium in a 1 L Erlenmeyer flask at 28 °C, with 70 g of rice (COFCO Group, Beijing, China), 0.1 g of corn flour (Macklin, Shanghai, China), 0.3 g of peptone (Solarbio, Beijing, China), 0.1 g of monosodium glutamate (Shandong Minghui Food Co., Ltd, Shandong, China), and 100 mL of filtered seawater.

### 3.3. Crude Extraction and Isolation

After fermentation for 30 days, the whole rice broth (from a total of 60 flasks) was extracted repeatedly with equivoluminal ethyl acetate (EtOAc) to yield 36 g of crude extract. The extract was then applied to silica gel vacuum liquid chromatography (VLC), and eluted with a petroleum ether (PE)–EtOAc and dichloromethane (DCM)–methanol (MeOH) gradient system to yield eight fractions (fractions 1–8). Fraction 4 (2.0 g), eluted with PE–EtOAc (2:1, *v*/*v*), was then subjected to reverse-phase column chromatography with a MeOH–H_2_O gradient system (from 1:9 to 10:0, *v*/*v*) to afford nine subfractions (fractions 4.1–4.9). Fraction 4.5 (26 mg) was further purified by CC on silica gel (DCM–MeOH gradient, from 20:1 to 10:1, *v*/*v*) to yield compound 1 (3.6 mg), and fraction 4.6 (0.3 g) was subjected to preparative thin layer chromatography (pTLC, DCM–MeOH, 20:1, *v*/*v*) to afford compound 2 (8.5 mg).

*Chryxanthone A* (compound **1**): yellowish oil; ${\left[\alpha \right]}_{D}^{25}$ −34.8 (*c* 0.10, MeOH); UV (MeOH) *λ*_max_ (log *ε*) 201 (3.88), 219 (3.96), 277 (3.46), 347 (3.12) nm; ECD (MeOH) 275 (+5.31), 305 (−2.59); ^1^H and ^13^C-NMR data (in DMSO-*d*_6_, 500 and 125 MHz), see Table 1; HRESIMS *m*/*z* 421.1653 [M − H]^−^ (calcd for C_25_H_25_O_6_, 421.1657).

*Chryxanthone B* (compound **2**): yellowish syrup; ${\left[\alpha \right]}_{D}^{25}$ −17.8 (*c* 0.11, MeOH); UV (MeOH) *λ*_max_ (log *ε*) 202 (3.61), 219 (3.62), 276 (3.34), 348 (2.96) nm; ECD (MeOH) 282 (+12.12), 328 (−8.31); ^1^H and ^13^C-NMR data (in DMSO-*d*_6_, 500 and 125 MHz), see Table 1; HRESIMS *m*/*z* 435.1814 [M − H]^−^ (calcd for C_26_H_27_O_6_, 435.1813).

### 3.4. Cytotoxicity Assay

The cytotoxicities against the A549, BT-549, HeLa, HepG2, MCF-7, and THP-1 cell lines were assayed by the Cell Counting Kit-8 (CCK-8) colorimetric method as described previously [22]. The percent inhibition of cell growth was calculated and expressed as the IC_50_ value. Epirubicin was used as a positive control.

## 4. Conclusions

In conclusion, two new benzophenone derivatives in structurally related xanthones, chryxanthone A (compound **1**) and B (compound **2**), were isolated and identified from the algal-derived endophytic fungus *P. chrysogenum* AD-1540. Both compounds represent a dihydropyran ring, which is unusual in naturally occurring benzophenone derivatives. Compounds **1** and **2** demonstrated weak to moderate cytotoxicities against some of human tumor cell lines.

## Figures and Tables

**Table 1:** ^1^H (500 MHz) and ^13^C-NMR (125 MHz) data of compounds **1** and **2** in DMSO-*d*_6_

	Compound 1		Compound 2	
Number	*δ*_H_ (mult, *J* in Hz)	*δ*_C_, type	*δ*_H_ (mult, *J* in Hz)	*δ*_C_, type
1		154.1, C		150.9, C
2		112.3, C		115.0, C
3	7.12, d (8.4)	132.4, CH	7.04, d (8.4)	128.1, CH
4	6.39, d (8.4)	108.6, CH	6.55, d (8.4)	104.3, CH
5	6.58, s	117.6, CH	6.52, s	118.7, CH
6		128.3, C		130.3, C
7		144.1, C		144.5, C
8		113.8, C		124.3, C
9		121.5, C		121.2, C
10		161.7, C		158.1, C
OH-10/ OCH_3_-10	12.20, s		3.69, s	56.3, CH_3_
11		146.9, C		150.0, C
OH-11	8.88, s		9.07, s	
12		126.4, C		127.0, C
13		200.6, C		198.1, C
14	6.26, d (9.8)	121.3, CH	6.31, d (9.8)	121.8, CH
15	5.44, d (9.8)	126.7, CH	5.51, d (9.8)	129.0, CH
16		76.7, C		76.6, C
17	0.98, s	27.2, CH_3_	1.13, s	28.2, CH_3_
18	0.92, s	26.6, CH_3_	0.79, s	26.6, CH_3_
19a 19b	4.18, m 4.13, m	62.8, CH_2_	4.26, dd (10.1, 3.0) 4.20, dd (11.0, 10.1)	63.1, CH_2_
20	2.34, br d (11.0)	44.4, CH	2.37, br d (11.0)	43.7, CH
21		142.1, C		142.6, C
22a 22b	4.83, s 4.61, s	111.6, CH_2_	4.87, s 4.65, s	111.9, CH_2_
23	1.70, s	22.1, CH_3_	1.76, s	22.5, CH_3_
24	2.10, s	16.0, CH_3_	2.07, s	16.9, CH_3_
25	4.53, br s	61.5, CH	4.59, br s	62.4, CH
OH-25	−		4.14, br s	

**Table 2:** Cytotoxicities (IC_50_, μM) of compounds **1** and **2**.

	A549 ^a^	BT-549 ^a^	HeLa ^a^	HepG2 ^a^	MCF-7 ^a^	THP-1 ^a^
**1**	41.7 ± 1.9	20.4 ± 1.2	23.5 ± 0.2	33.6 ± 1.4	46.4 ± 1.2	>50
**2**	20.4 ± 0.9	>50	>50	>50	>50	41.1 ± 0.5
Epirubicin ^b^	7.2 ± 0.11	5.3 ± 0.02	2.9 ± 0.04	4.6 ± 0.11	5.2 ± 0.02	5.5 ± 0.04

^a^ A549, human lung adenocarcinoma epithelial cell line; BT-549, human breast cancer cell line; HeLa, human cervix carcinoma cell line; HepG2, human liver carcinoma cell line; MCF-7, human breast adenocarcinoma cell line; NCI-H460, human monocytic cell line. ^b^ Positive control.

## Supplementary Materials

The following are available online at http://www.mdpi.com/1420-3049/23/12/3378/s1. Scheme S1: Proposed biosynthetic pathways to compounds 1,2; Figure S1: HRESIMS spectrum of 1; Figure S2: ^1^H-NMR spectrum of 1; Figure S3: ^13^C-NMR spectrum of 1; Figure S4: COSY spectrum of 1; Figure S5: HSQC spectrum of 1; Figure S6: HMBC spectrum of 1; Figure S7: NOESY spectrum of 1; Figure S8: HRESIMS spectrum of 2; Figure S9: ^1^H-NMR spectrum of 2; Figure S10: ^13^C-NMR and DEPT spectra of 2; Figure S11: COSY spectrum of 2; Figure S12: HSQC spectrum of 2; Figure S13: HMBC spectrum of 2; Figure S14: NOESY spectrum of 2; Figure S15: Structure and population of the low-energy B3LYP/6-31G(d) conformers (>2%) of (20*S*,25*S*)-1; Figure S16: Structure and population of the low-energy B3LYP/6-31G(d) conformers (>2%) of (20*S*,25*S*)-2; Table S1: Cartesian coordinates of the low-energy conformers (≥2%) of 1; Table S2: Cartesian coordinates of the low-energy conformers (≥2%) of 2.

## References

[1] Blunt J.W., Carroll A.R., Copp B.R., Davis R.A., Keyzers R.A., Prinsep M.R. (2018). Marine natural products. Nat. Prod. Rep..

[2] Chen M., Shao C.L., Meng H., She Z.G., Wang C.Y. (2014). Anti-respiratory syncytial virus prenylated dihydroquinolone derivatives from the gorgonian-derived fungus *Aspergillus* sp. XS-20090B15. J. Nat. Prod..

[3] Imhoff J.F. (2016). Natural products from marine fungi—Still an underrepresented resource. Mar. Drugs.

[4] Qi B., Liu X., Mo T., Li S.S., Wang J., Shi X.P., Wang X.H., Zhu Z.X., Zhao Y.F., Jin H.W. (2017). Nitric oxide inhibitory polyketides from *Penicillium chrysogenum* MT-12, an endophytic fungus isolated from *Huperzia serrata*. Fitoterapia.

[5] Guo W.Q., Li D., Peng J.X., Zhu T.J., Gu Q.Q., Li D.H. (2015). Penicitols A–C and penixanacid A from the mangrove-derived *Penicillium chrysogenum* HDN11-24. J. Nat. Prod..

[6] Wang J.F., Zhou L.M., Chen S.T., Yang B., Liao S.R., Kong F.D., Lin X.P., Wang F.Z., Zhou X.F., Liu Y.H. (2018). New chlorinated diphenyl ethers and xanthones from a deep-sea-derived fungus *Penicillium chrysogenum* SCSIO 41001. Fitoterapia.

[7] Peng J.X., Zhang X.M., Du L., Wang W., Zhu T.J., Gu Q.Q., Li D.H. (2014). Sorbicatechols A and B, antiviral sorbicillinoids from the marine-derived fungus *Penicillium chrysogenum* PJX-17. J. Nat. Prod..

[8] Li Z.X., Wang X.F., Ren G.W., Yuan X.L., Deng N., Ji G.X., Li W., Zhang P. (2018). Prenylated diphenyl ethers from the marine algal-derived endophytic fungus *Aspergillus tennesseensis*. Molecules.

[9] Zhao D.L., Wang D., Tian X.Y., Cao F., Li Y.Q., Zhang C.S. (2018). Anti-phytopathogenic and cytotoxic activities of crude extracts and secondary metabolites of marine-derived fungi. Mar. Drugs.

[10] Chexal K.K., Fouweather C., Holker J.S.E., Simpson T.J., Young K. (1974). The biosynthesis of fungal metabolites. Part III. Structure of shamixanthone and tajixanthone, metabolites of *Aspergillus variecolor*. J. Chem. Soc. Perkin Trans. 1.

[11] Moosophon P., Kanokmedhakul S., Kanokmedhakul K., Soytong K. (2009). Prenylxanthones and a bicyclo[3.3.1]nona-2,6-diene derivative from the fungus *Emericella rugulosa*. J. Nat. Prod..

[12] Wu X., Fang L.Z., Liu F.L., Pang X.J., Qin H.L., Zhao T., Xu L.L., Yang D.F., Yang X.L. (2017). New prenylxanthones, polyketide hemiterpenoid pigments from the endophytic fungus *Emericella* sp. XL029 and their anti-agricultural pathogenic fungal and antibacterial activities. RSC Adv..

[13] Zhu A., Yang M.Y., Zhang Y.H., Shao C.L., Wang C.Y., Hu L.D., Cao F., Zhu H.J. (2018). Absolute configurations of 14,15-hydroxylated prenylxanthones from a marine-derived *Aspergillus* sp. fungus by chiroptical methods. Sci. Rep..

[14] Zhu A., Zhang X.W., Zhang M., Li W., Ma Z.Y., Zhu H.J., Cao F. (2018). Aspergixanthones I–K, new anti-*Vibrio* prenylxanthones from the marine-derived fungus *Aspergillus* sp. ZA-01. Mar. Drugs.

[15] Wu Q., Wu C., Long H., Chen R., Liu D., Proksch P., Guo P., Lin W. (2015). Varioxiranols A–G and 19-*O*-methyl-22-methoxypre-shamixanthone, PKS and hybrid PKS-derived metabolites from a sponge-associated *Emericella variecolor* fungus. J. Nat. Prod..

[16] Ishida M., Hamasaki T., Hatsuda Y., Fukuyama K., Tsukihara T., Katsube Y. (1976). Epishamixanthone, a new metabolite from *Aspergillus rugulosus*. Agr. Biol. Chem..

[17] (2013). Gaussian 09.

[18] Sanchez J.F., Entwistle R., Hung J.H., Yaegashi J., Jain S., Chiang Y.M., Wang C.C.C., Oakley B.R. (2011). Genome-based deletion analysis reveals the prenyl xanthone biosynthesis pathway in *Aspergillus nidulans*. J. Am. Chem. Soc..

[19] Holker J.S.E., Lapper R.D., Simpson T.J. (1974). The biosynthesis of fungal metabolites. Part IV. Tajixanthone: ^13^C nuclear magnetic resonance spectrum and feedings with [1-^13^C]- and [2-^13^C]-acetate. J. Chem. Soc. Perkin Trans. 1.

[20] Kralj A., Kehraus S., Krick A., Eguereva E., Kelter G., Maurer M., Wortmann A., Fiebig H.H., König G.M. (2006). Arugosins G and H: prenylated polyketides from the marine-derived fungus *Emericella nidulans* var. *acristata*. J. Nat. Prod..

[21] Wang S., Li X.M., Teuscher F., Li D.L., Diesel A., Ebel R., Proksch P., Wang B.G. (2006). Chaetopyranin, a benzaldehyde derivative, and other related metabolites from *Chaetomium globosum*, an endophytic fungus derived from the marine red alga *Polysiphonia urceolata*. J. Nat. Prod..

[22] Yuan X.L., Zhang P., Liu X.M., Du Y.M., Hou X.D., Cheng S., Zhang Z.F. (2017). Cytological assessments and transcriptome profiling demonstrate that evodiamine inhibits growth and induces apoptosis in a renal carcinoma cell line. Sci. Rep..

