# Supplementary material for: Benzophenone Derivatives from an Algal-Endophytic Isolate of Penicillium chrysogenum and Their Cytotoxicity
Source: Molecules. 2018 Dec 19;23(12):3378. doi: 10.3390/molecules23123378 (PMC6321059; doi:10.3390/molecules23123378)
Supplement: Supplementary file 1 [file molecules-23-03378-s001.pdf]

Table of Contents

Scheme S1. Proposed biosynthetic pathways to compounds **1**–**2**

Figure S1. HRESIMS spectrum of compound **1**

Figure S2. ^{1}H NMR (500 MHz, DMSO-d_6) spectrum of compound **1**

Figure S3. ^{13}C NMR (125 MHz, DMSO-d_6) spectrum of compound **1**

Figure S4. COSY spectrum of compound **1**

Figure S5. HSQC spectrum of compound **1**

Figure S6. HMBC spectrum of compound **1**

Figure S7. NOESY spectrum of compound **1**

Figure S8. HRESIMS spectrum of compound **2**

Figure S9. ^{1}H NMR (500 MHz, DMSO-d_6) spectrum of compound **2**

Figure S10. ^{13}C NMR (125 MHz, DMSO-d_6) and DEPT spectra of compound **2**

Figure S11. COSY spectrum of compound **2**

Figure S12. HSQC spectrum of compound **2**

Figure S13. HMBC spectrum of compound **2**

Figure S14. NOESY spectrum of compound **2**

Figure S15. Structure and population of the low-energy B3LYP/6-31G(d) conformers (>2%) of (20*S*,25*S*)-**1**.

Figure S16. Structure and population of the low-energy B3LYP/6-31G(d) conformers (>2%) of (20*S*,25*S*)-**2**.

Table S1. Cartesian coordinates of the low-energy conformers (≥2%) of **1**

Table S2. Cartesian coordinates of the low-energy conformers (≥2%) of **2**

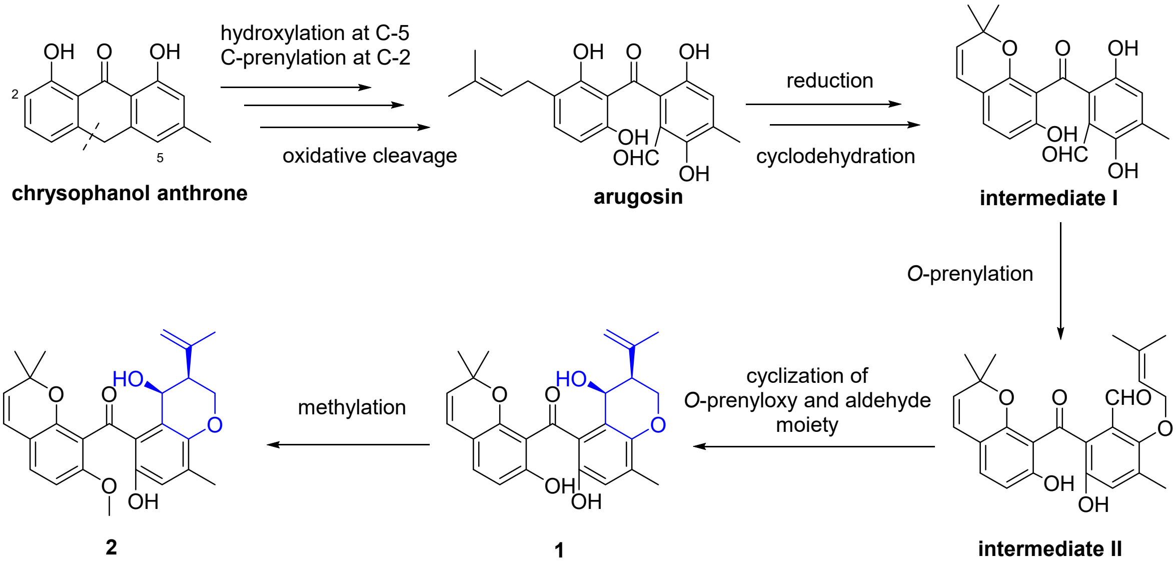

Scheme S1 Proposed biosynthetic pathways to compounds **1**–**2.**

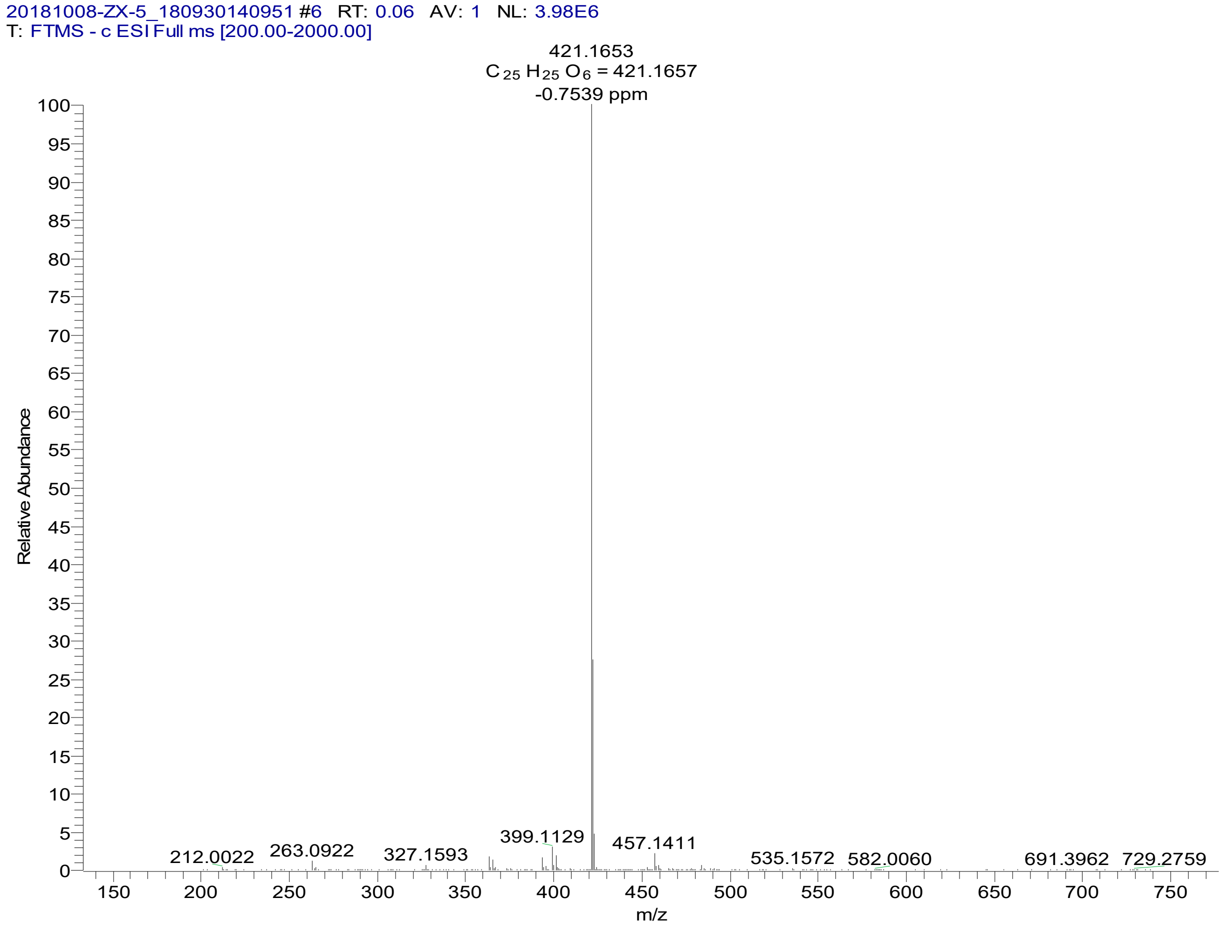

Figure S1. HRESIMS spectrum of compound **1.**

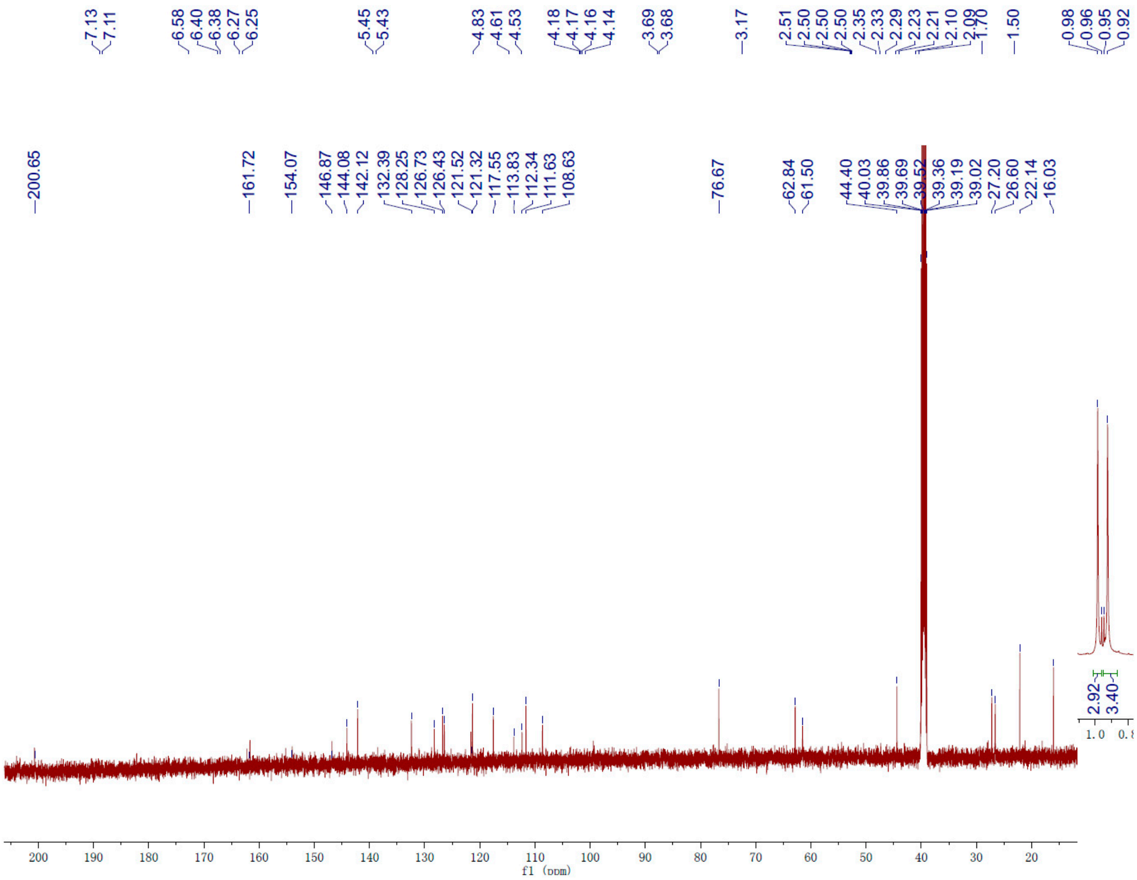

Figure S3. ^{13}C NMR (125 MHz, DMSO-d_6) spectrum of compound **1.**

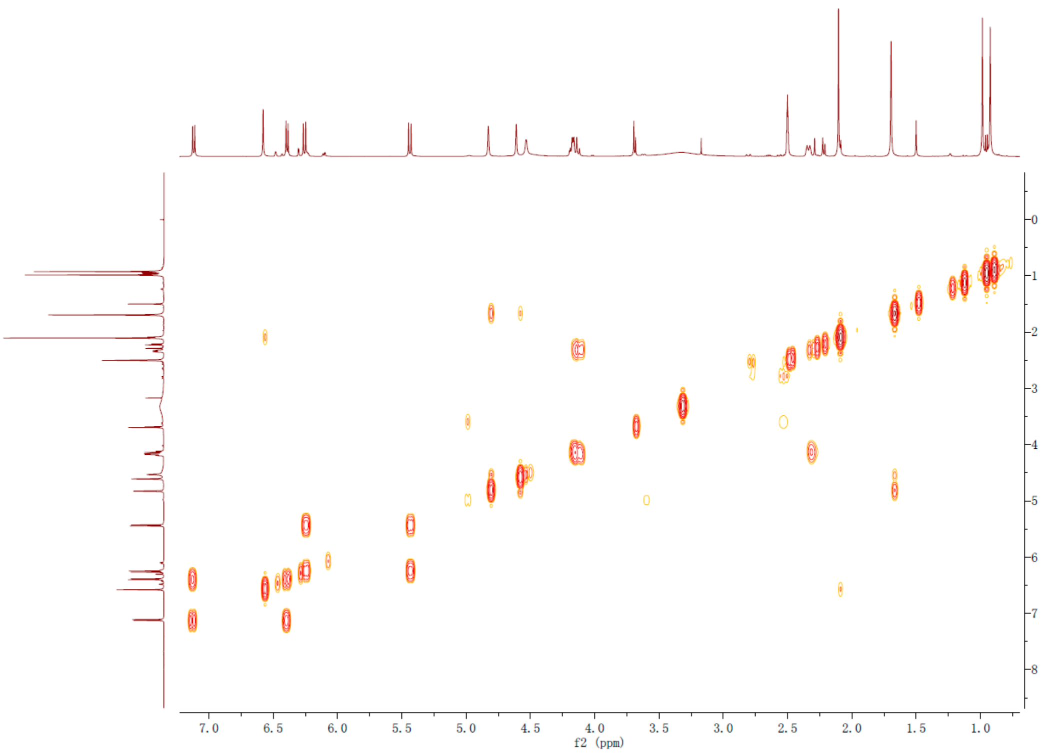

Figure S4. COSY spectrum of compound **1.**

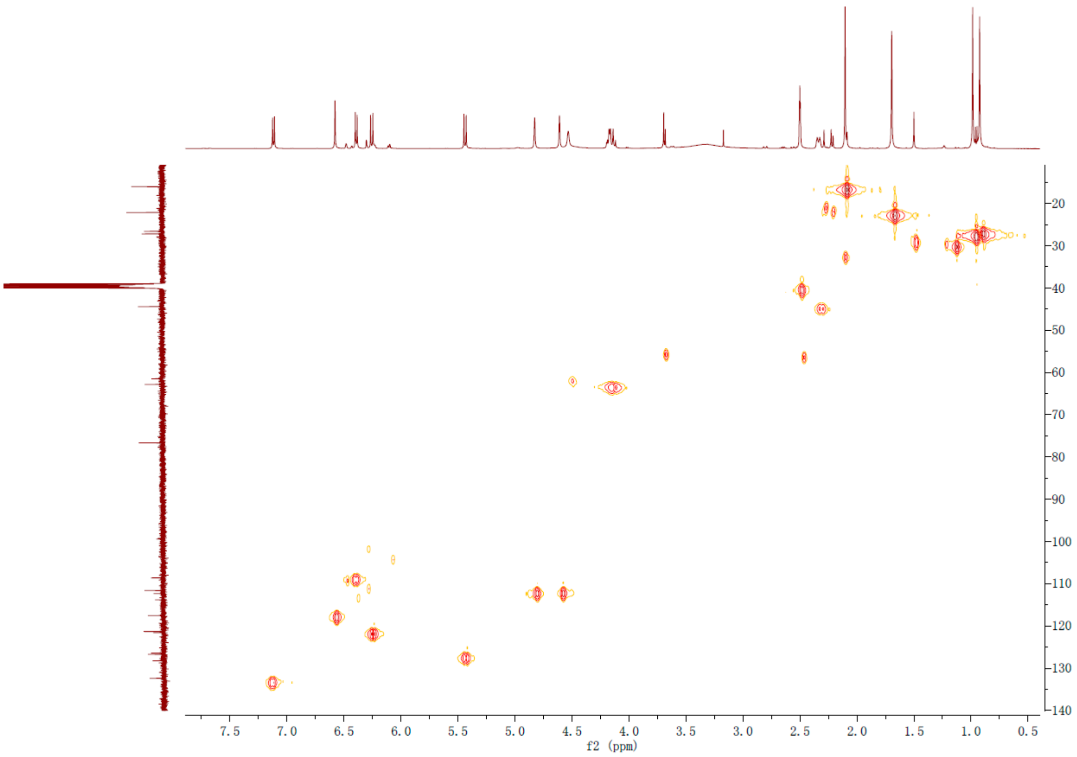

Figure S5. HSQC spectrum of compound **1.**

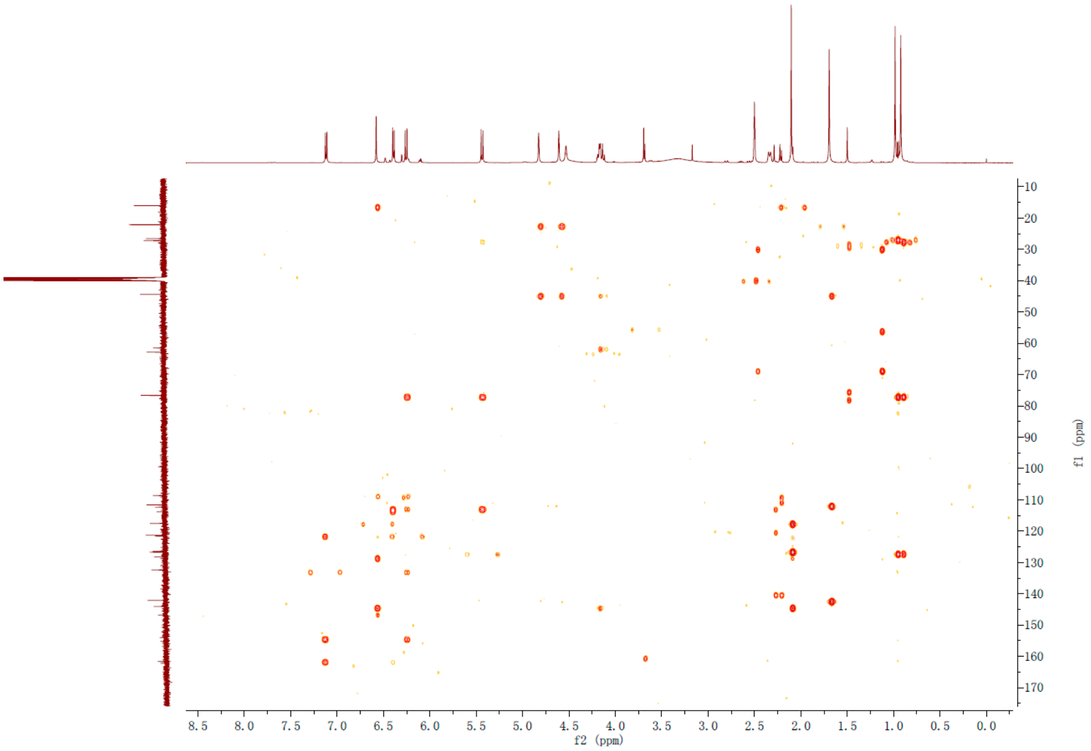

Figure S6. HMBC spectrum of compound **1.**

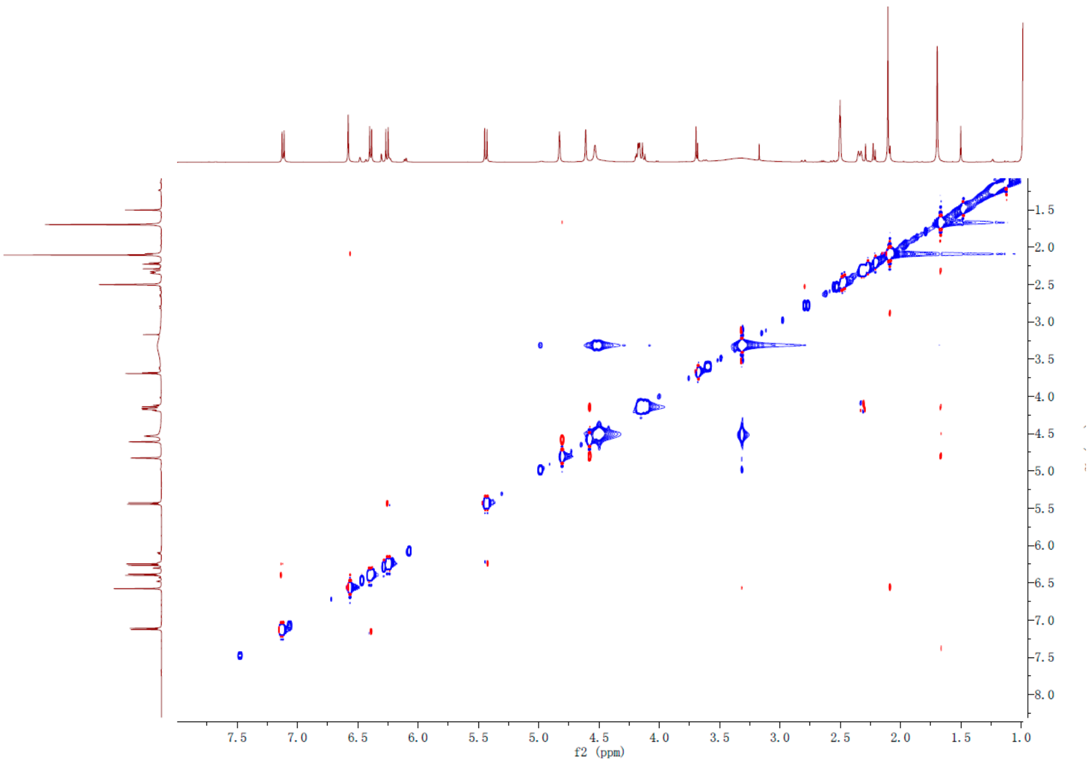

Figure S7. NOESY spectrum of compound **1.**

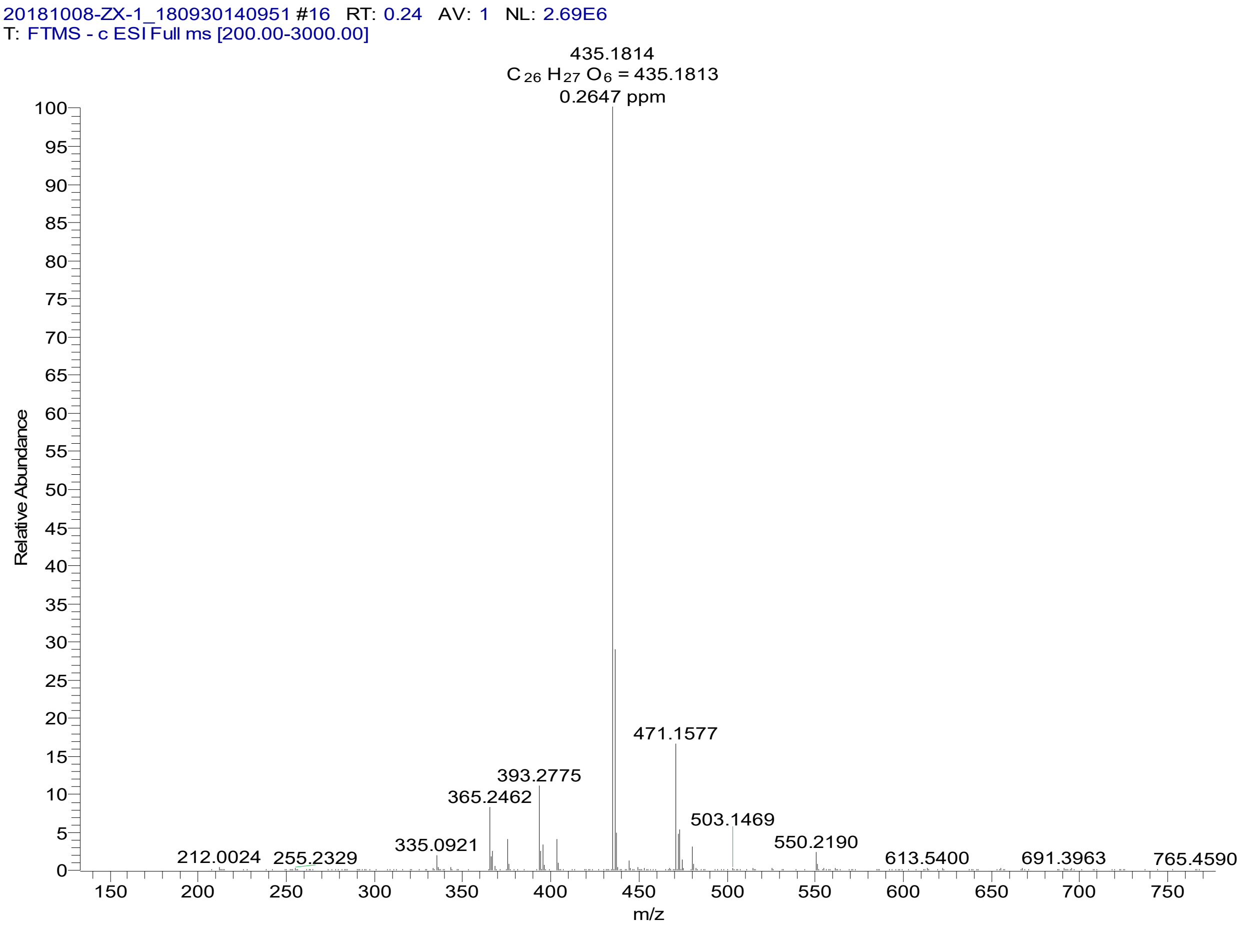

Figure S8. HRESIMS spectrum of compound **2.**

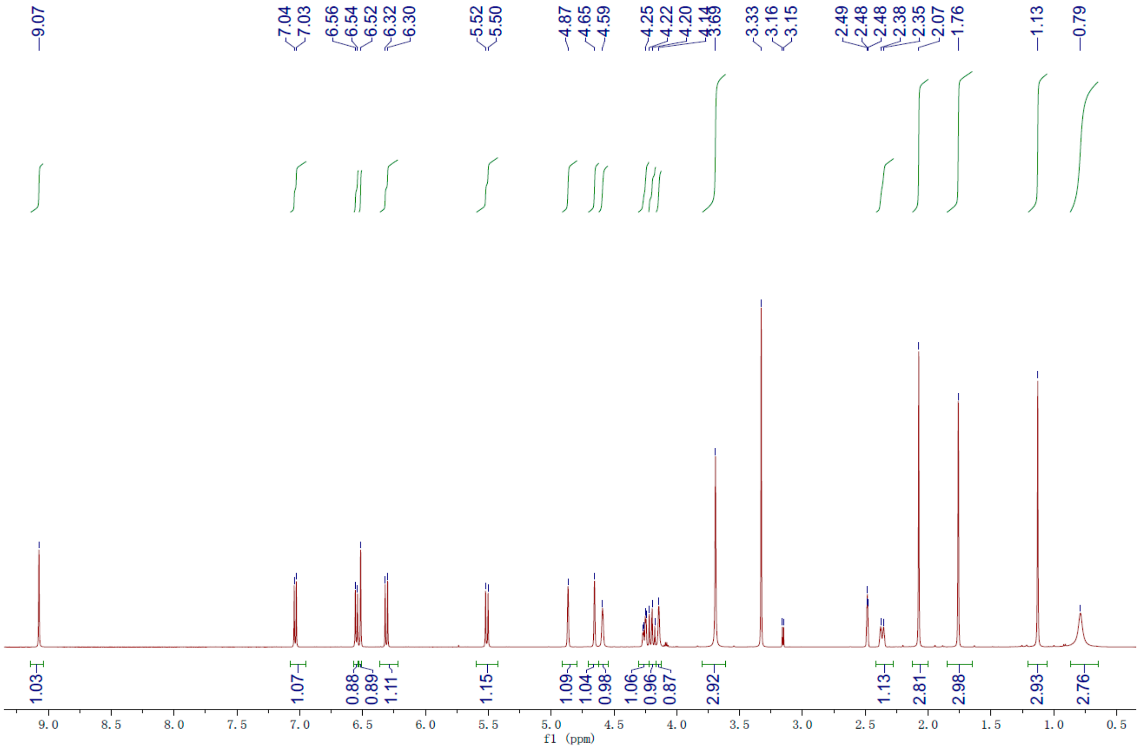

Figure S9. ^{1}H NMR (500 MHz, DMSO-d_6) spectrum of compound **2.**

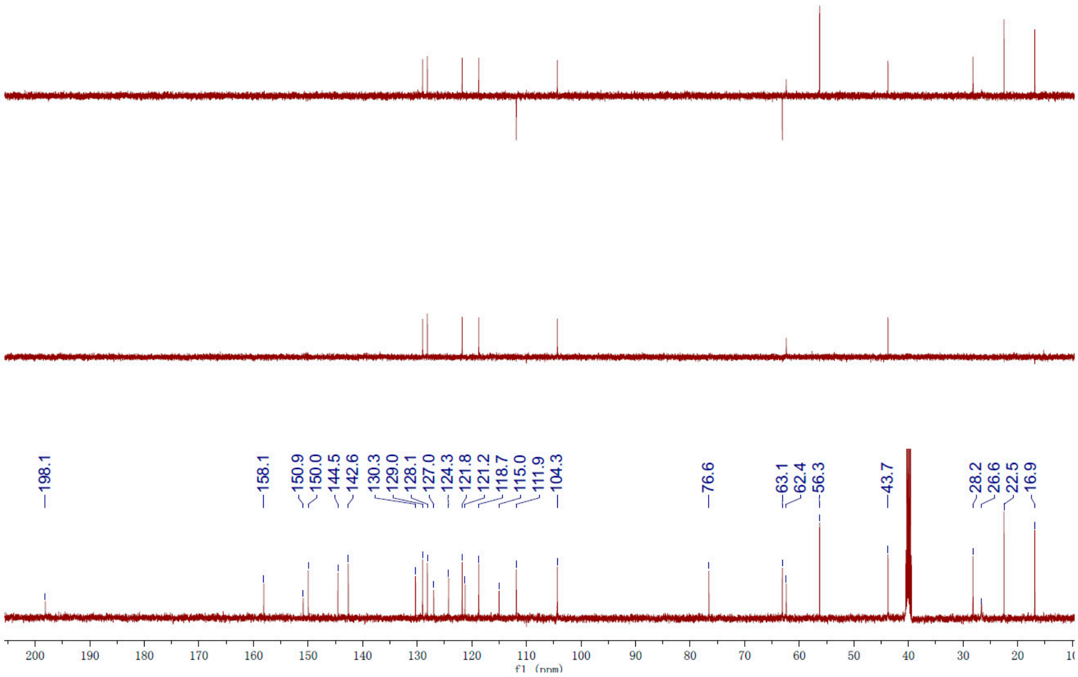

Figure S10. ^{13}C NMR (125 MHz, DMSO-d_6) and DEPT spectra of compound **2.**

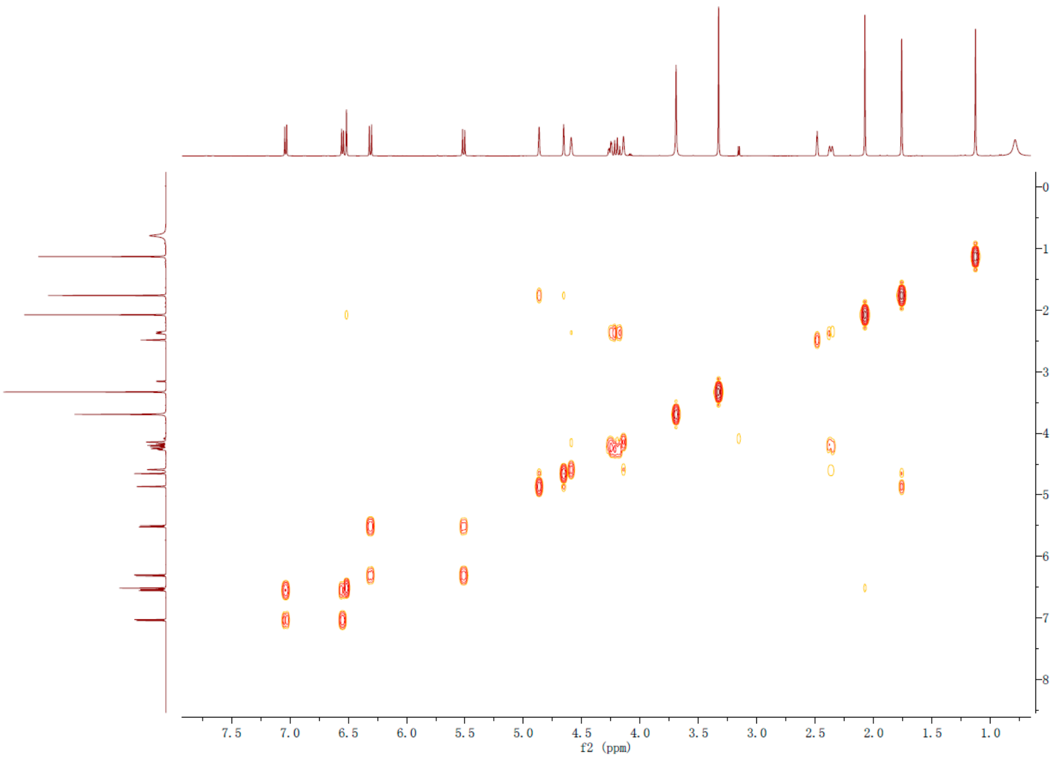

Figure S11. COSY spectrum of compound **2.**

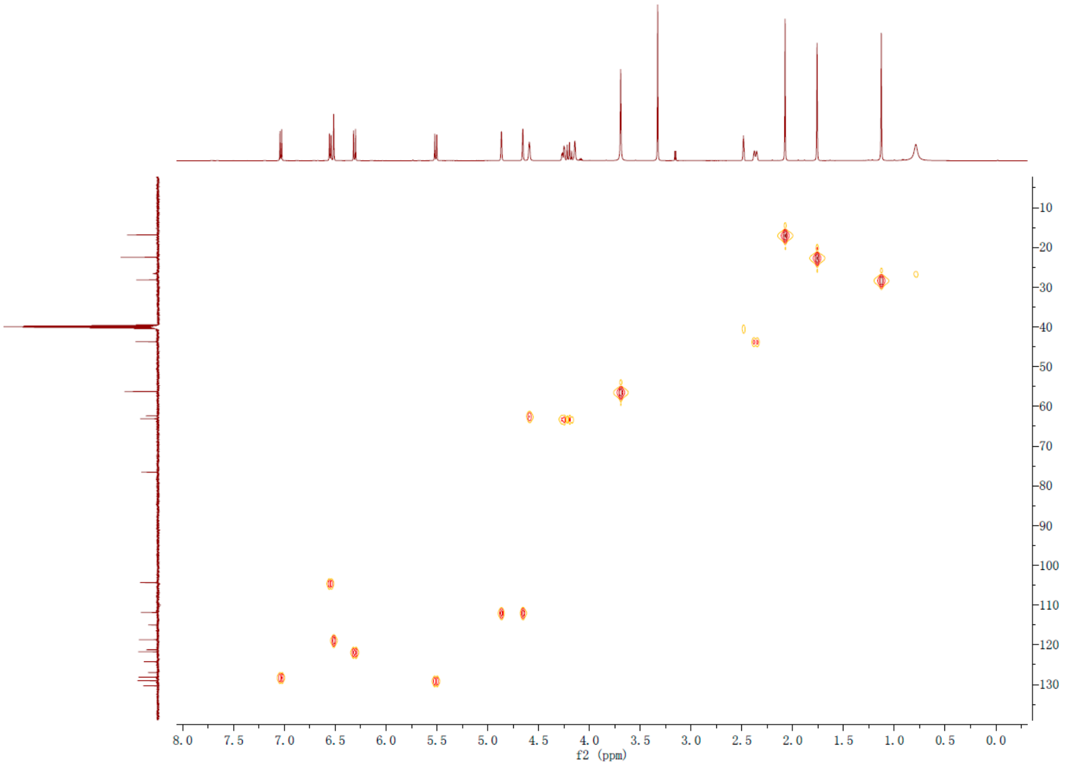

Figure S12. HSQC spectrum of compound **2.**

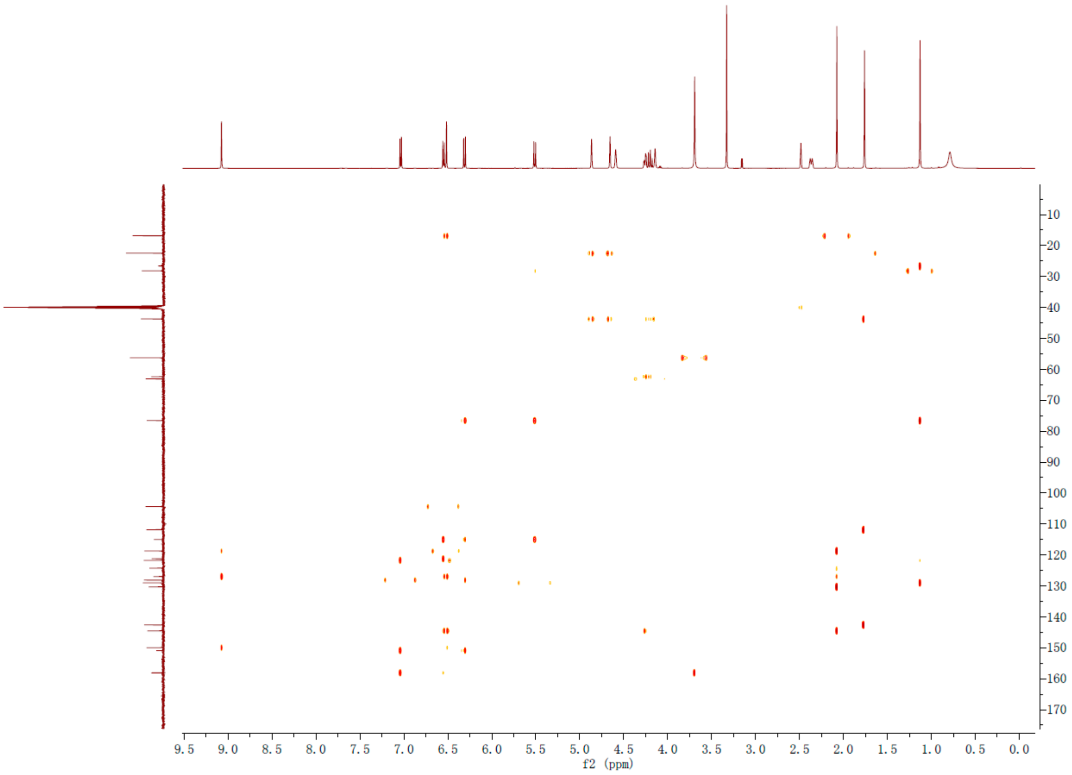

Figure S13. HMBC spectrum of compound **2.**

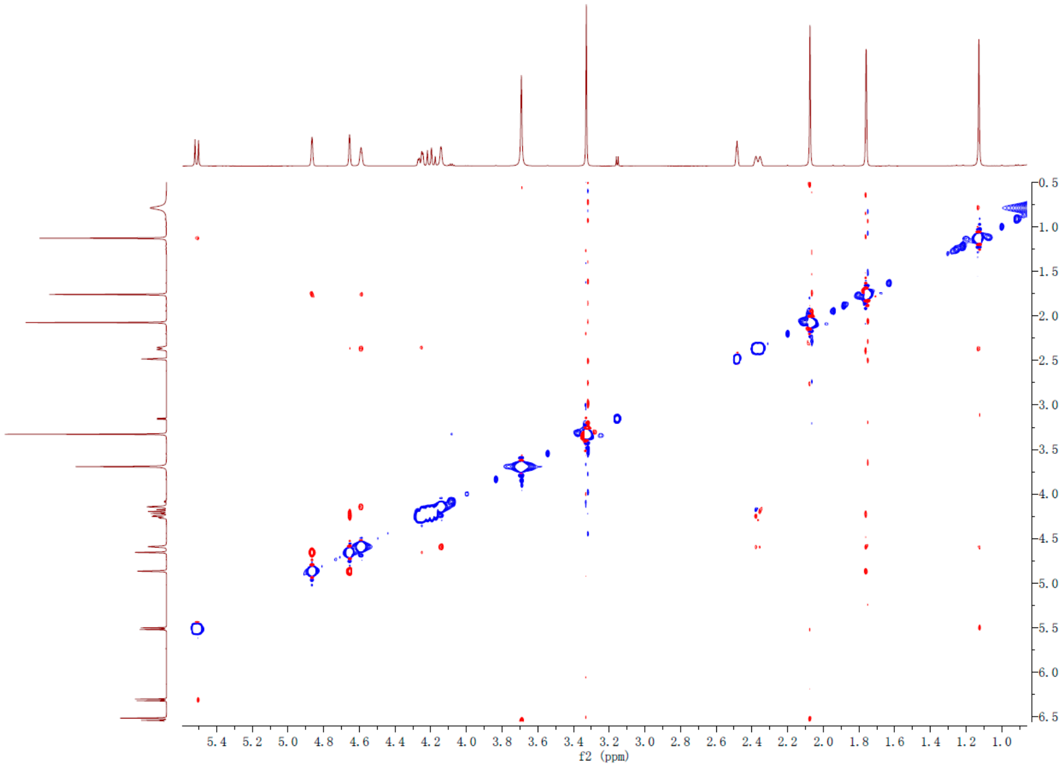

Figure S14. NOESY spectrum of compound **2.**

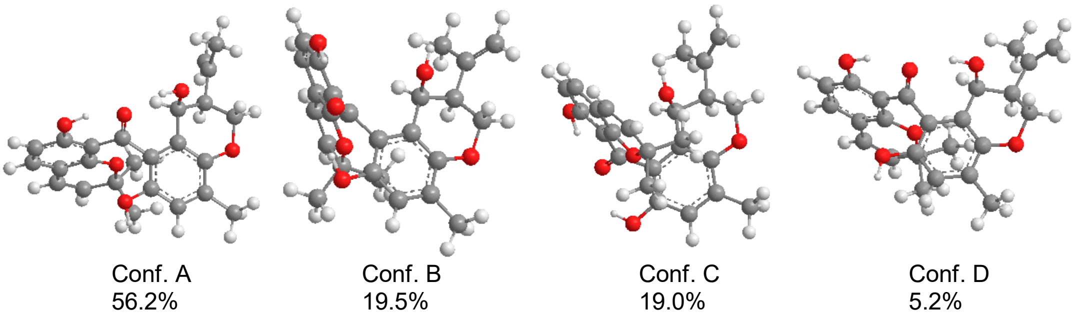

Figure S15. Structure and population of the low-energy B3LYP/6-31G(d) conformers (>2%) of (20*S*,25*S*)-**1**.

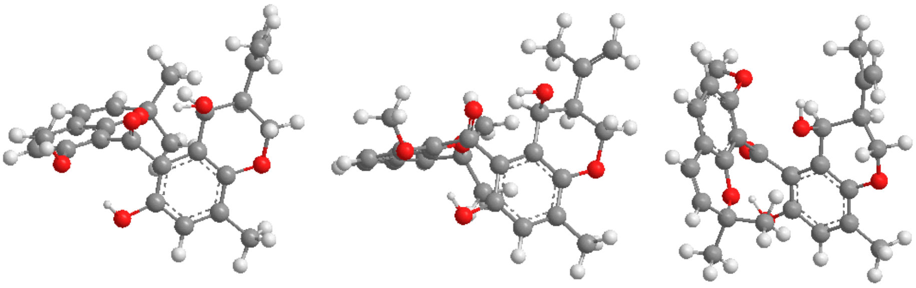

Conf. A 76.2% | Conf. B 13.3% | Conf. C 10.4%

Figure S16. Structure and population of the low-energy B3LYP/6-31G(d) conformers (>2%) of (20*S*,25*S*)-**2**.

Table S1. Cartesian coordinates of the low−energy conformers (≥2%) of **1.**

(20*S*,25*S*)−**1**,Conf. A		Standard Orientation (Ångstroms)		
No.	Atom	X	Y	Z
1	C	−3.76162	−0.57921	0.86732
2	C	−4.5329	−1.62064	0.33156
3	C	−4.03741	−2.47303	−0.64583
4	C	−2.72756	−2.30604	−1.10515
5	C	−1.91494	−1.21445	−0.64677
6	C	−2.46466	−0.36806	0.36272
7	O	−1.66483	0.57329	0.90795
8	C	−2.21728	1.6945	1.69893
9	C	−3.47041	1.26945	2.42395
10	C	−4.20358	0.23238	1.99577
11	C	−0.57854	−1.04168	−1.22014
12	C	0.2056	0.23372	−1.16002
13	O	−0.04086	−1.99305	−1.84572
14	C	1.50757	0.23973	−0.60734
15	C	2.26267	1.41876	−0.64969
16	C	1.76592	2.58898	−1.26038
17	C	0.49504	2.54405	−1.83163
18	C	−0.28638	1.38585	−1.7832
19	C	−2.51984	2.85085	0.73462
20	C	−1.09436	2.05998	2.67163
21	O	−2.27894	−3.19996	−2.01042
22	O	−1.53075	1.32621	−2.36373
23	C	2.59743	3.84575	−1.295
24	O	3.50661	1.53206	−0.07827
25	C	4.16756	0.32016	0.32464
26	C	3.19632	−0.62096	1.03956
27	C	2.08281	−1.00862	0.04813
28	O	2.62251	−1.90358	−0.93927
29	C	3.87695	−1.81037	1.70753
30	C	3.472	−2.20528	2.92453
31	C	5.00578	−2.51066	0.98624
32	H	−5.53548	−1.78038	0.72174
33	H	−4.62945	−3.29376	−1.03722
34	H	−3.77396	1.87213	3.27624
35	H	−5.12875	−0.04682	2.49496
36	H	0.10435	3.42987	−2.32864
37	H	−3.25883	2.54688	−0.01343
38	H	−1.6078	3.17538	0.22349
39	H	−2.92787	3.69961	1.29535
40	H	−0.18423	2.32204	2.1224
41	H	−1.39372	2.9222	3.27728
42	H	−0.87446	1.22368	3.34299
43	H	−1.31424	−3.01269	−2.15267
44	H	−1.76111	2.19074	−2.74116
45	H	2.82717	4.19826	−0.28187
46	H	3.55892	3.67243	−1.79304
47	H	2.07213	4.64536	−1.82564

48	H	4.59913	−0.15492	−0.5644
49	H	4.97602	0.64633	0.98278
50	H	2.70294	−0.03878	1.82884
51	H	1.29249	−1.52834	0.6066
52	H	1.88359	−2.10579	−1.54601
53	H	3.92511	−3.0582	3.42546
54	H	2.67252	−1.69436	3.45773
55	H	5.36759	−3.36495	1.56764
56	H	5.85612	−1.83426	0.82409
57	H	4.68494	−2.8622	0.00033

(20*S*,25*S*)-**1**,Conf. B		Standard Orientation (Ångstroms)		
No.	Atom	X	Y	Z
1	C	3.09089	0.55046	0.75576
2	C	3.98575	1.10041	−0.17318
3	C	3.8452	0.88516	−1.53823
4	C	2.77579	0.11798	−2.01042
5	C	1.77796	−0.38665	−1.11507
6	C	1.99836	−0.19966	0.27925
7	O	1.10296	−0.72181	1.14922
8	C	1.51438	−1.02733	2.53454
9	C	2.44613	0.0465	3.04604
10	C	3.20406	0.75514	2.1963
11	C	0.63324	−1.12532	−1.67508
12	C	−0.68915	−1.27989	−1.01193
13	O	0.78176	−1.70942	−2.7831
14	C	−1.43491	−0.20186	−0.45635
15	C	−2.66815	−0.46768	0.14236
16	C	−3.16613	−1.78669	0.26937
17	C	−2.44293	−2.81921	−0.31219
18	C	−1.24761	−2.57541	−0.99935
19	C	2.20527	−2.39984	2.52588
20	C	0.20587	−1.07054	3.32312
21	O	2.71875	−0.10103	−3.34318
22	O	−0.65605	−3.6564	−1.59446
23	C	−4.47294	−2.0417	0.97398
24	O	−3.47756	0.5097	0.67114
25	C	−3.23015	1.85865	0.24461
26	C	−1.74399	2.17656	0.35672
27	C	−0.96602	1.2346	−0.59754
28	O	−1.14585	1.58838	−1.98184
29	C	−1.37943	3.63554	0.13214
30	C	−2.05896	4.43832	−0.70332
31	C	−0.17619	4.12713	0.90179
32	H	4.82042	1.69427	0.19198
33	H	4.56212	1.27894	−2.25126
34	H	2.50769	0.1843	4.12251
35	H	3.90842	1.50224	2.55521
36	H	−2.81888	−3.83805	−0.2768

37	H	3.101	−2.38186	1.89608
38	H	1.52191	−3.1705	2.15166
39	H	2.51002	−2.66818	3.54366
40	H	−0.47395	−1.81367	2.89435
41	H	0.41099	−1.34734	4.36276
42	H	−0.28739	−0.09356	3.31512
43	H	2.02573	−0.78967	−3.49804
44	H	−0.0146	−3.32419	−2.25464
45	H	−5.29499	−1.4983	0.49275
46	H	−4.71336	−3.10877	0.97156
47	H	−4.43587	−1.69753	2.01473
48	H	−3.59009	1.97051	−0.78542
49	H	−3.83256	2.4854	0.90463
50	H	−1.43095	1.90516	1.375
51	H	0.09567	1.3129	−0.35493
52	H	−0.92209	2.53238	−2.07675
53	H	−1.76199	5.47466	−0.84815
54	H	−2.93299	4.10719	−1.25771
55	H	−0.36799	4.08863	1.98282
56	H	0.08085	5.15661	0.63382
57	H	0.70268	3.4949	0.71762

(20*S*,25*S*)-**1**,Conf. C		Standard Orientation (Ångstroms)		
No.	Atom	X	Y	Z
1	C	2.98973	1.01866	−0.92025
2	C	3.4935	2.18492	−0.33028
3	C	3.16894	2.5495	0.97279
4	C	2.29562	1.74802	1.71121
5	C	1.66537	0.60756	1.11849
6	C	2.08571	0.22516	−0.18629
7	O	1.59603	−0.91891	−0.70278
8	C	2.29804	−1.61179	−1.8008
9	C	2.95871	−0.61332	−2.72169
10	C	3.31109	0.6031	−2.28071
11	C	0.67915	−0.14285	1.90154
12	C	−0.43281	−0.93215	1.30115
13	O	0.76237	−0.14377	3.15906
14	C	−1.35917	−0.39188	0.36859
15	C	−2.39308	−1.20647	−0.10534
16	C	−2.50417	−2.56454	0.27637
17	C	−1.6031	−3.06126	1.21071
18	C	−0.61068	−2.24819	1.76767
19	C	1.2063	−2.40763	−2.51733
20	C	3.33962	−2.54429	−1.16224
21	O	2.04988	2.11062	2.99238
22	O	0.18341	−2.81555	2.73217
23	C	−3.61427	−3.42104	−0.27537
24	O	−3.40581	−0.75684	−0.91425
25	C	−3.37963	0.60756	−1.37003

26	C	−2.81184	1.53639	−0.3014
27	C	−1.35496	1.0774	−0.01596
28	O	−0.53385	1.26192	−1.17597
29	C	−2.87883	3.01537	−0.64641
30	C	−2.83219	3.48074	−1.9057
31	C	−2.97727	3.94602	0.53955
32	H	4.1773	2.80682	−0.90362
33	H	3.60029	3.42882	1.44003
34	H	3.17633	−0.94187	−3.73478
35	H	3.82678	1.31045	−2.92649
36	H	−1.68578	−4.08821	1.5563
37	H	0.45373	−1.73737	−2.94401
38	H	0.71421	−3.0946	−1.82122
39	H	1.64938	−2.99508	−3.32877
40	H	2.8505	−3.26444	−0.49638
41	H	3.87402	−3.09802	−1.94257
42	H	4.07355	−1.96963	−0.5875
43	H	1.5681	1.36314	3.42342
44	H	0.60072	−2.09819	3.24715
45	H	−3.55183	−4.43906	0.11999
46	H	−4.59776	−3.00916	−0.01772
47	H	−3.572	−3.47048	−1.37012
48	H	−2.78381	0.64898	−2.28793
49	H	−4.41761	0.85025	−1.60625
50	H	−3.38321	1.38008	0.62424
51	H	−0.94813	1.66978	0.81327
52	H	−0.6153	2.19593	−1.44309
53	H	−2.86498	4.54898	−2.10778
54	H	−2.77818	2.83016	−2.77428
55	H	−3.91916	3.78192	1.08064
56	H	−2.93422	4.99571	0.23332
57	H	−2.16796	3.76548	1.25964

(20*S*,25*S*)-**1**,Conf. D		Standard Orientation (Ångstroms)		
No.	Atom	X	Y	Z
1	C	−3.73825	−0.55496	0.85554
2	C	−4.52831	−1.56215	0.28283
3	C	−4.05287	−2.37866	−0.73431
4	C	−2.74437	−2.21056	−1.19712
5	C	−1.91279	−1.15078	−0.6998
6	C	−2.44283	−0.33977	0.34859
7	O	−1.62629	0.56768	0.92584
8	C	−2.16013	1.66424	1.76296
9	C	−3.4092	1.22278	2.48531
10	C	−4.15889	0.21291	2.02219
11	C	−0.57754	−0.97401	−1.27459
12	C	0.2264	0.28567	−1.16386
13	O	−0.05829	−1.90593	−1.94348
14	C	1.52815	0.25165	−0.61137

15	C	2.29653	1.42203	−0.60299
16	C	1.81432	2.62444	−1.16021
17	C	0.54393	2.6198	−1.73397
18	C	−0.25044	1.46932	−1.73754
19	C	−2.46403	2.85802	0.84552
20	C	−1.0237	1.9847	2.73564
21	O	−2.3154	−3.0717	−2.14273
22	O	−1.49474	1.44873	−2.32089
23	C	2.661	3.87133	−1.13767
24	O	3.54068	1.4913	−0.02702
25	C	4.1928	0.25025	0.29423
26	C	3.22776	−0.71574	0.97643
27	C	2.08472	−1.0307	−0.00867
28	O	2.60248	−1.90066	−1.02864
29	C	3.87598	−1.9647	1.55529
30	C	5.17544	−2.2583	1.40285
31	C	2.96104	−2.85991	2.35939
32	H	−5.52971	−1.72483	0.67483
33	H	−4.65954	−3.17378	−1.15506
34	H	−3.6961	1.79228	3.36571
35	H	−5.08168	−0.0773	2.51948
36	H	0.16366	3.53146	−2.19074
37	H	−3.20998	2.58592	0.09211
38	H	−1.55442	3.19874	0.34074
39	H	−2.86459	3.68635	1.44115
40	H	−0.11688	2.25747	2.18617
41	H	−1.30865	2.82786	3.37413
42	H	−0.80478	1.12342	3.37499
43	H	−1.34901	−2.89293	−2.28408
44	H	−1.71503	2.331	−2.66137
45	H	3.6188	3.7105	−1.64692
46	H	2.14413	4.70182	−1.62767
47	H	2.898	4.17205	−0.10968
48	H	4.59845	−0.18174	−0.62863
49	H	5.01772	0.53287	0.95031
50	H	2.7638	−0.1737	1.81559
51	H	1.28048	−1.54898	0.52966
52	H	1.8599	−2.06439	−1.64253
53	H	5.60298	−3.1491	1.85782
54	H	5.85959	−1.64331	0.82529
55	H	2.439	−2.29371	3.14264
56	H	3.52097	−3.67042	2.83628
57	H	2.18705	−3.3142	1.72709

Table S2. Cartesian coordinates of the low−energy conformers (≥2%) of **2.**

(20*S*,25*S*)−**2**,Conf. A		Standard Orientation (Ångstroms)		
No.	Atom	X	Y	Z
1	C	3.62695	1.48921	0.17477
2	C	4.71452	0.70375	−0.21677
3	C	4.54105	−0.55464	−0.80104
4	C	3.24293	−1.02044	−1.01426
5	C	2.12046	−0.25519	−0.64801
6	C	2.32391	0.98857	−0.04375
7	O	1.22449	1.7446	0.23325
8	C	1.30941	2.79229	1.26581
9	C	2.6679	3.45722	1.2322
10	C	3.74792	2.82598	0.74653
11	C	0.72008	−0.68741	−1.03465
12	C	−0.18986	−1.29862	−0.02922
13	O	0.35467	−0.45119	−2.18968
14	C	−1.56136	−0.88894	0.03532
15	C	−2.38482	−1.42913	1.02601
16	C	−1.89947	−2.36255	1.97303
17	C	−0.57949	−2.77636	1.86807
18	C	0.27356	−2.27576	0.87644
19	C	1.05727	2.1345	2.63176
20	C	0.19442	3.77554	0.90763
21	O	2.93345	−2.26532	−1.51297
22	O	1.52606	−2.82611	0.85501
23	C	−2.8101	−2.90351	3.04359
24	O	−3.70196	−1.07861	1.18971
25	C	−4.33012	−0.3615	0.11549
26	C	−3.42362	0.77099	−0.36257
27	C	−2.12561	0.15755	−0.92354
28	O	−2.39297	−0.42118	−2.21122
29	C	−4.11018	1.74449	−1.31432
30	C	−3.89496	3.06257	−1.18378
31	C	−5.03958	1.19915	−2.37444
32	C	3.99373	−3.10713	−1.99629
33	H	5.72192	1.08206	−0.06135
34	H	5.40446	−1.14943	−1.07333
35	H	2.73694	4.45382	1.66103
36	H	4.72838	3.29672	0.75858
37	H	−0.18691	−3.52794	2.54734
38	H	1.81928	1.37742	2.84518
39	H	0.06995	1.65952	2.65072
40	H	1.09616	2.89038	3.42429
41	H	0.36866	4.2168	−0.07898
42	H	0.15519	4.5818	1.64789
43	H	−0.77439	3.26584	0.90064
44	H	1.95802	−2.75308	−0.02108
45	H	−3.19133	−2.09729	3.68223
46	H	−3.68573	−3.39776	2.6062

47	H	−2.28252	−3.6245	3.67453
48	H	−4.55497	−1.06658	−0.69426
49	H	−5.26704	0.01157	0.53492
50	H	−3.12578	1.33748	0.52951
51	H	−1.39722	0.96787	−1.04274
52	H	−1.51087	−0.65372	−2.56332
53	H	−4.36043	3.78364	−1.85251
54	H	−3.24448	3.46471	−0.40961
55	H	−4.53074	0.45685	−2.99714
56	H	−5.4146	2.00439	−3.01459
57	H	−5.90932	0.7005	−1.92479
58	H	4.6797	−3.37288	−1.18563
59	H	3.50236	−4.0027	−2.37612
60	H	4.53584	−2.60673	−2.80441

(20*S*,25*S*)-**2**,Conf. B		Standard Orientation (Ångstroms)		
No.	Atom	X	Y	Z
1	C	3.59338	1.48408	0.25309
2	C	4.68961	0.71986	−0.15595
3	C	4.52972	−0.51786	−0.78644
4	C	3.23672	−0.98396	−1.02851
5	C	2.10602	−0.23809	−0.64767
6	C	2.29574	0.98395	0.00327
7	O	1.18922	1.72343	0.29765
8	C	1.25909	2.72455	1.37665
9	C	2.61158	3.40258	1.37805
10	C	3.7	2.79923	0.87573
11	C	0.71175	−0.66645	−1.05937
12	C	−0.20165	−1.3086	−0.07705
13	O	0.35406	−0.39966	−2.21012
14	C	−1.57481	−0.90546	−0.00693
15	C	−2.39691	−1.46836	0.97118
16	C	−1.91081	−2.41972	1.89975
17	C	−0.58984	−2.82846	1.78741
18	C	0.26278	−2.3043	0.80762
19	C	1.0082	2.00365	2.71059
20	C	0.13623	3.71248	1.05868
21	O	2.93994	−2.21391	−1.56975
22	O	1.51685	−2.85045	0.77532
23	C	−2.8219	−2.98396	2.95778
24	O	−3.71394	−1.122	1.13765
25	C	−4.34401	−0.40654	0.06303
26	C	−3.45696	0.74381	−0.39735
27	C	−2.13878	0.1597	−0.94405
28	O	−2.39432	−0.38323	−2.24891
29	C	−4.11828	1.71687	−1.3626
30	C	−5.3546	1.54127	−1.85102
31	C	−3.30313	2.93819	−1.71963
32	C	4.01094	−3.0358	−2.06362

33	H	5.69309	1.09821	0.02296
34	H	5.39949	−1.09767	−1.07052
35	H	2.66942	4.38248	1.84529
36	H	4.6765	3.27685	0.91231
37	H	−0.19603	−3.59339	2.45087
38	H	1.77737	1.24588	2.89361
39	H	0.02597	1.51788	2.70349
40	H	1.03582	2.72379	3.5361
41	H	0.31014	4.20025	0.09414
42	H	0.087	4.48374	1.83478
43	H	−0.82804	3.19526	1.02437
44	H	1.95158	−2.75215	−0.09681
45	H	−2.29503	−3.7192	3.57267
46	H	−3.20259	−2.19197	3.61436
47	H	−3.69787	−3.46745	2.50926
48	H	−4.54846	−1.10827	−0.75541
49	H	−5.28966	−0.05567	0.47936
50	H	−3.18506	1.31717	0.50322
51	H	−1.40766	0.97064	−1.03158
52	H	−1.50966	−0.60373	−2.60231
53	H	−5.80073	2.27691	−2.51682
54	H	−5.96662	0.67312	−1.62302
55	H	−2.96268	3.46546	−0.81841
56	H	−3.88556	3.63804	−2.32712
57	H	−2.4038	2.66763	−2.2879
58	H	4.56047	−2.50955	−2.85008
59	H	4.68814	−3.32108	−1.25219
60	H	3.52947	−3.92265	−2.47524

(20*S*,25*S*)−**2**,Conf. C		Standard Orientation (Ångstroms)		
Number	Atom	X	Y	Z
1	C	−1.1233	1.1245	0.6412
2	C	−0.3412	1.3214	−0.5037
3	C	−0.6344	0.6361	−1.6837
4	C	−1.7223	−0.2369	−1.7276
5	C	−2.4982	−0.4565	−0.5821
6	C	−2.2047	0.2344	0.6029
7	O	−3.0331	0.0502	1.6772
8	C	−2.5594	0.4335	2.983
9	C	−1.4981	1.5098	2.9715
10	C	−0.8239	1.8485	1.8684
11	C	−3.6436	−1.3874	−0.6369
12	C	−3.8091	−2.4886	0.3468
13	O	−4.5511	−1.1883	−1.4456
14	C	−2.8055	−3.4409	0.6289
15	C	−3.0664	−4.4147	1.6144
16	C	−4.2996	−4.4595	2.2923
17	C	−5.2965	−3.5388	1.9595
18	C	−5.0582	−2.5786	0.9844

19	C	−2.0267	−0.8262	3.6762
20	C	−3.7939	0.9486	3.7328
21	O	−1.9391	−0.9591	−2.8729
22	O	−6.1093	−1.7502	0.6835
23	C	−4.5728	−5.5099	3.3327
24	O	−2.1836	−5.4156	1.9405
25	C	−0.857	−5.2791	1.4246
26	C	−0.8658	−4.8888	−0.0562
27	C	−1.5028	−3.487	−0.1267
28	O	−0.6313	−2.5206	0.4606
29	C	0.4804	−4.9641	−0.7844
30	C	1.674	−5.0671	−0.1742
31	C	0.3997	−4.9432	−2.2908
32	C	−2.6237	−0.1595	−3.8401
33	H	0.5054	2.0037	−0.4833
34	H	−0.0087	0.7762	−2.5609
35	H	−1.2682	2.0052	3.9108
36	H	−0.056	2.6128	1.8759
37	H	−6.2689	−3.5715	2.4444
38	H	−1.1713	−1.2443	3.1326
39	H	−2.7908	−1.6118	3.698
40	H	−1.7115	−0.6234	4.7055
41	H	−4.2162	1.8285	3.2324
42	H	−3.5624	1.2197	4.7687
43	H	−4.5871	0.1915	3.7445
44	H	−5.9067	−1.3189	−0.1758
45	H	−4.6185	−6.4993	2.8668
46	H	−3.7893	−5.5031	4.0975
47	H	−5.526	−5.3317	3.8417
48	H	−0.308	−4.5658	2.0521
49	H	−0.3766	−6.2558	1.5536
50	H	−1.5422	−5.5944	−0.5624
51	H	−1.6741	−3.2221	−1.1753
52	H	0.1662	−2.4854	−0.0963
53	H	2.5976	−5.1309	−0.7443
54	H	1.7828	−5.0884	0.9055
55	H	−0.2474	−5.7507	−2.6481
56	H	1.3813	−5.0773	−2.758
57	H	−0.0043	−3.9891	−2.6426
58	H	−3.4919	0.3536	−3.4115
59	H	−1.9393	0.5681	−4.2888
60	H	−2.9789	−0.8247	−4.6327
